# Minor Loops in Major Folds: Enhancer–Promoter Looping, Chromatin Restructuring, and Their Association with Transcriptional Regulation and Disease

**DOI:** 10.1371/journal.pgen.1005640

**Published:** 2015-12-03

**Authors:** Navneet Matharu, Nadav Ahituv

**Affiliations:** 1 Department of Bioengineering and Therapeutic Sciences, University of California, San Francisco, San Francisco, California, United States of America; 2 Institute for Human Genetics, University of California, San Francisco, San Francisco, California, United States of America; University College London, UNITED KINGDOM

## Abstract

The organization and folding of chromatin within the nucleus can determine the outcome of gene expression. Recent technological advancements have enabled us to study chromatin interactions in a genome-wide manner at high resolution. These studies have increased our understanding of the hierarchy and dynamics of chromatin domains that facilitate cognate enhancer–promoter looping, defining the transcriptional program of different cell types. In this review, we focus on vertebrate chromatin long-range interactions as they relate to transcriptional regulation. In addition, we describe how the alteration of boundaries that mark discrete regions in the genome with high interaction frequencies within them, called topological associated domains (TADs), could lead to various phenotypes, including human diseases, which we term as “TADopathies.”

## DNA Folding and Transcriptional Gene Regulation

DNA is bound by a variety of histone proteins, noncoding RNAs, and transcription factors, collectively called chromatin. Packaging of chromatin within a eukaryotic nucleus can bring distant loci together, which may affect their transcriptional state. Numerous experiments have shown that the juxtaposition of two distant loci is not random, and their functional outcome depends upon the nature of interacting loci and the histone modifications they harbor. This information is encoded in the sequence of the loci that determines the transcription factors that bind to the DNA. Histone-modifying enzymes and complexes play a crucial role in regulating chromatin accessibility (for an in-depth review of how chromatin modifications regulate gene expression, refer to citations [1–3]). Understanding the determinants that organize this cell type-specific spatial arrangement of long DNA molecules is crucial in order to comprehend how genomes are regulated.

Much of our understanding of eukaryotic gene expression has been gained by characterizing gene regulatory elements. Apart from the promoters that are quintessential for gene expression, distal regulatory elements control gene expression in a spatiotemporal manner. These elements include the following: “repressors,” which can inhibit gene promoters; “insulators,” which obliterate enhancer–promoter interaction when present between them, thus preventing non-cognate enhancer–promoter crosstalk; “barriers,” which can also be considered as another type of insulator, which stop heterochromatin spreading and maintain borders between euchromatin and heterochromatin regions [4]; and “enhancers,” which target gene promoters to activate gene transcription. Amongst these elements, the widest functional diversity between tissues has been seen for enhancers, suggesting that these elements have an important role in determining tissue specificity [5].

How an enhancer identifies its target promoter in three-dimensional nuclear space still remains largely unknown. Several mechanisms have been proposed that explain enhancer–promoter interactions based on direct contact and non-contact models: (1) juxtaposition of enhancer and promoter by looping out the intervening region; (2) enhancer tracking over the intervening region to identify its target promoter; (3) chromatin modifications over large regions regulated through chromatin modifiers that act as a “link” between the enhancer and promoter [6,7]. To date, the most investigated contact model for enhancer–promoter interaction is the looping model, which has been supported by numerous reports including recent biochemical techniques that can identify chromatin interactions in a genome-wide manner.

## Methods to Detect Gene Regulatory Interactions

Several methods have been developed that take advantage of the postulated looping mechanism to identify enhancer–promoter interactions. DNA-fluorescence in situ hybridization (DNA-FISH) can be used to visualize specific DNA interactions of two loci that are more than 100 kb apart or on different chromosomes [8,9]. The two interacting loci in question are probed with nearly 10 kb- to 100 kb-long DNA fragments labeled with different fluorophores on fixed cells. The proximity distance of the two signals within a nucleus is measured, and interactions can be compared to a non-interacting cell type or tissue. This technique allows for the visualization of interactions in individual cells, thus also providing the ability to observe cell-to-cell variations. For example, the sonic hedgehog (*SHH*) limb enhancer, which resides over 900 kb away from *SHH*, was shown to physically interact with its promoter in developing mouse limbs using DNA-FISH [10]. DNA-FISH was also used in mouse limb buds to show the chromatin looping interaction between the 5′*HoxD* complex and the global control region (GCR) module in the distal posterior limb bud [11]. However, the limitation of this technique is its resolution. DNA fragments less than 100 kb apart are difficult to visualize separately by this technique. Additionally, only a few different loci can be studied at a time.

Chromosome Conformation Capture (3C) and its derivative methods have become the major biochemical approaches to studying chromatin interactions. These methods are all based on the principles of 3C, whereby chromatin is first cross-linked using formaldehyde so that DNA regions within spatial proximity are linked together with protein complexes. DNA is then fragmented, followed by ligation at a specific dilution set to facilitate intramolecular ligation of DNA regions. In conventional 3C, locus-specific primers are used for PCR-based detection of the ligated products [12]. 4C is used in order to study interactions with respect to a specific locus (a promoter, for example) where inverse PCR from the chosen locus is employed to detect novel interacting loci [13]. To characterize interactions over a specific locus, 5C can be used, which takes advantage of a multitude of primers that cover a region [14].

With advancement in massively parallel sequencing technologies, it has become possible to study interactions among regulatory elements on a genome-wide scale using Hi-C [15]. The initial steps of Hi-C are similar up to the chromatin fragmentation step, after which DNA overhangs are end-filled and biotinylated linkers are ligated before intramolecular chromatin ligation. Using streptavidin beads, DNA fragments having biotinylated ligation junctions are purified and sequenced using massively parallel sequencing. One commonly used modification of Hi-C is chromatin immunoprecipitation interaction assay with paired end tagging (ChIA-PET). ChIA-PET adds a chromatin immunoprecipitation (ChIP) step that allows for the enrichment of specific interactions and, thus, higher resolution [16]. The use of RNA Polymerase II antibody in this ChIP step, for example, can allow for the enrichment of promoter interactions [17,18]. A modification of Hi-C that uses sequence capture technologies, termed Capture-C [19], can also allow for the identification of specific interactions. For an in depth review of 3C literature, see [20,21].

It is important to note that these 3C-associated techniques also have several caveats. They require formaldehyde crosslinking of the sample that provides only a snapshot of the interactions [22–24]. These looping studies should, thus, be corroborated with a separate set of functional data to support any functional consequence. In addition, it is understood that current chromosome capture techniques generate both soluble and insoluble fractions of chromatin complexes, which then are subjected to proximity ligation protocols, therefore not providing a full picture of interactions within an active chromatin hub [22]. One such example for this caveat is the study of the *HoxD* locus using 5C and DNA-FISH [24]. In the wild-type state, the interaction results were comparable between techniques, whereas in the Polycomb repressive complex 1 null background, in which the *HoxD* complex gets de-repressed, DNA-FISH showed more open chromatin conformations as compared to 5C data, which demonstrated a more compact *HoxD* cluster. Therefore, it is important to be cautious to interpret these interactions as bona fide functional interactions.

## Topological Associated Domains

**Fig 1:**
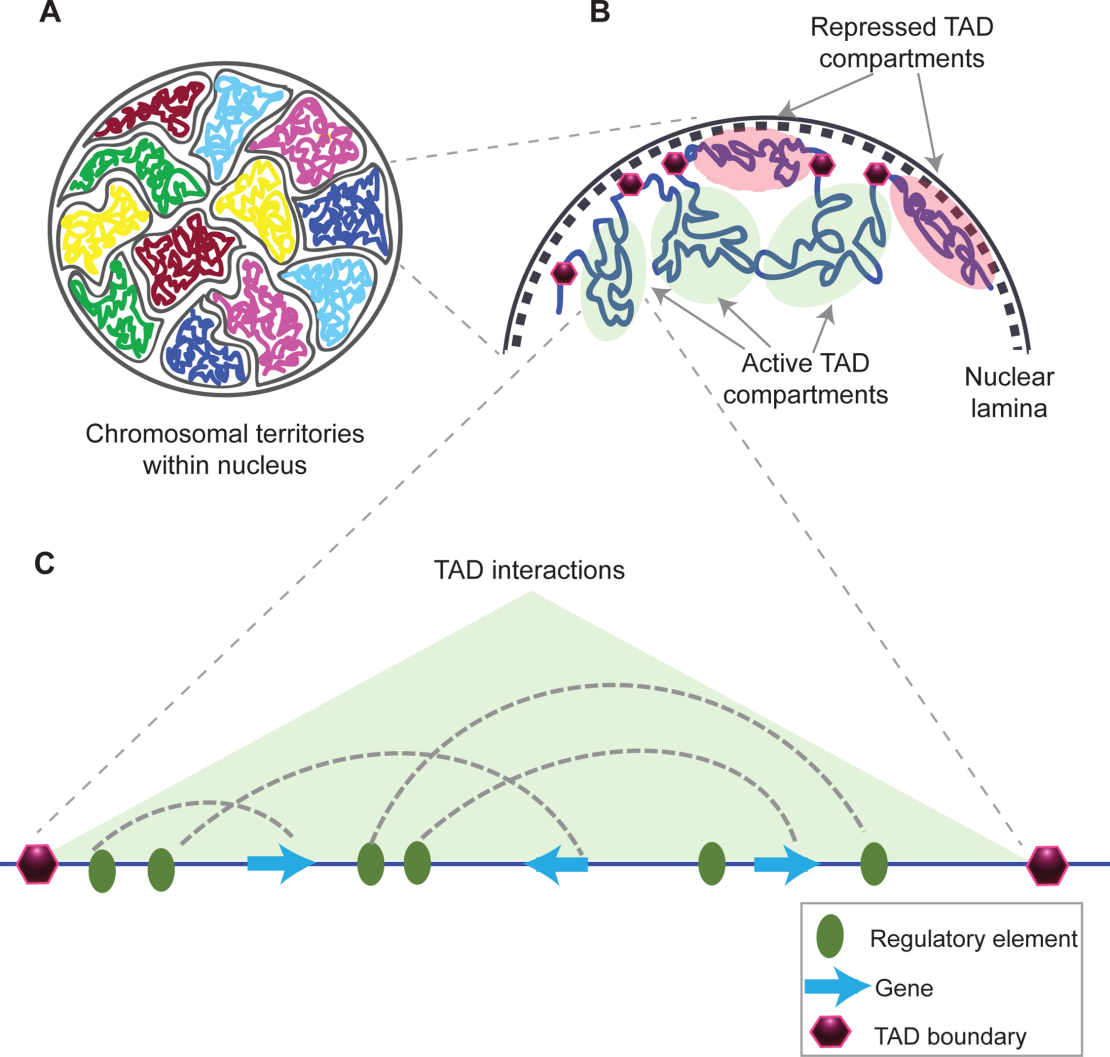
Structural organization of chromatin. (A) Chromosomes within an interphase diploid eukaryotic nucleus are found to occupy specific nuclear spaces, termed chromosomal territories [25]. (B) Each chromosome is subdivided into topological associated domains (TAD) as found in Hi-C studies. TADs with repressed transcriptional activity tend to be associated with the nuclear lamina (dashed inner nuclear membrane and its associated structures), while active TADs tend to reside more in the nuclear interior [32,33]. Each TAD is flanked by regions having low interaction frequencies, as determined by Hi-C, that are called TAD boundaries (purple hexagon) [29]. (C) An example of an active TAD with several interactions between distal regulatory elements and genes within it.

Each chromosome occupies a microscopically visible territory within the interphase nucleus that, in turn, is divided into topological domains (Fig 1A) [25,26]. Through the above-mentioned biochemical techniques, genome-wide DNA interaction maps have been generated for different cell types and organisms. These maps have revealed discrete regions in the genome that have high interaction frequencies within them, termed topological associated domains (TADs), and are bordered by low interaction regions called TAD boundaries (Fig 1B and 1C). These domains are classified on the basis of the frequency of DNA interactions within hundreds of kilobases of a DNA region. Coordination of gene expression depends upon the interplay of several regulatory elements and their cognate promoters within a TAD. TADs also subdivide the genome into structural domains of coordinated replicating regions. It has been observed that TADs correlate with the regions of uniform replication timing, also called constant timing regions (CTRs). TAD boundaries have been shown to be associated with the timing transition regions (TTRs), segments of DNA that lie between two differentially replicating CTRs, when flanked by early replicating CTRs [27]. TAD boundaries are suggested to have barrier activity, which can stop heterochromatin spreading from neighboring domains [28]. Histone 3 lysine 27 tri-methylation (H3K27me3) modification, SUZ12 polycomb repressive complex 2 subunit (Suz12), RNA Polymerase II (RNAPolII), and CCCTC-binding factor (CTCF) binding are enriched at TAD boundaries [27]. TAD boundaries were also shown to have a high occupancy of “architectural-insulator proteins” [29–31]. In addition, comparison between human and mouse TAD boundaries showed strong similarities, suggesting that these can be conserved between organisms [28]. TADs constitute discrete structural units across the genome, with numerous gene regulatory interactions occurring within them. How this structural framework translates into the functional outcome of gene expression largely depends on the local chromatin environment and biocompatibility of transcription factors that regulate gene expression.

## Chromatin De-condensation and Locus Repositioning

Chromatin condensation is directly linked to transcription, with closed chromatin representing transcriptionally repressed regions and open chromatin having an active transcription state. DNA regions that are tethered to inner nuclear periphery via interactions with the lamin B1 protein are called lamina-associated domains (LADs) [32]. These are generally 0.1 to 10 megabases in size and are marked by low gene expression and repressive histone marks. In contrast, actively transcribing genes tend to leave their chromosome territory and LADs to associate with RNAPolII foci toward the interior of the nucleus (Fig 1B). For an in-depth review on chromatin compartmentalization literature, refer to [34–38].

**Fig 2:**
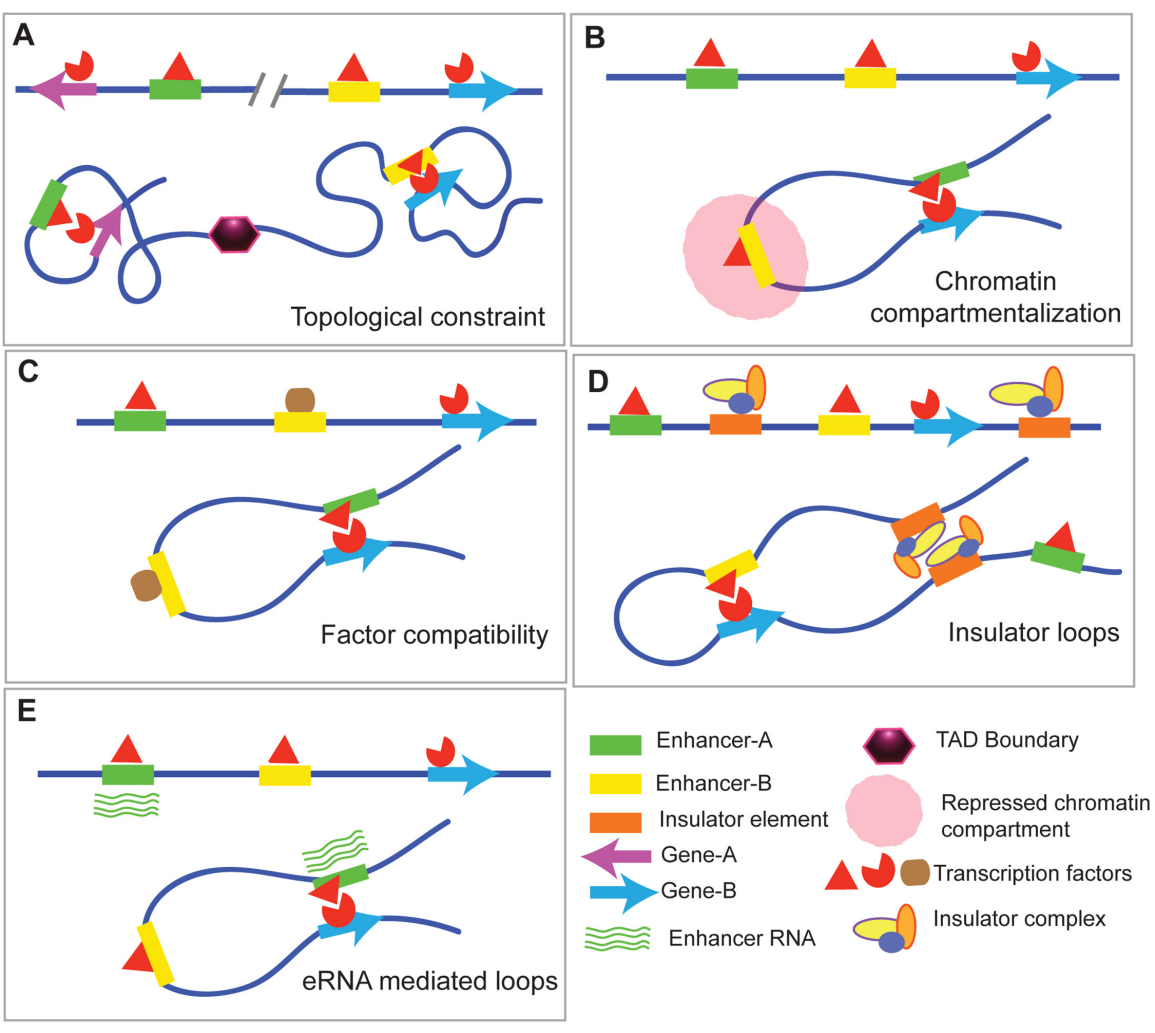
Mechanisms defining enhancer–promoter recognition. (A) TAD boundaries pose spatial constraint, allowing for specific enhancer–promoter interactions. (B) Enhancer activity could be obscured by its localization to repressive chromatin compartments. (C) Enhancer–promoter targeting can depend upon the binding compatibility of the transcription factors bound to two interacting loci, termed biochemical compatibility. (D) Insulator elements bound by insulator factors facilitate specific enhancer interaction and eliminate nonspecific enhancer–promoter crosstalk. (E) Noncoding RNA or enhancer RNA (eRNA) transcribed from the enhancer can define its interaction with the target promoter.

How a locus repositions and what factors cause this relocation are still open questions. It has been shown that induction of locus repositioning takes place upon differentiation and development and that it involves large-scale epigenetic modifications. Differentiation of embryonic stem cells (ESC) to neural precursor cells (NPC) leads to upregulation of many fate specification genes. A subset of upregulated genes exhibits loss of laminB1 association [39]. Pleiotrophin (*Ptn*), sex-determining region Y (SRY)-box 6 (*Sox6*), and neuropilin1 (*Nrp1*) genes show the largest loss of laminB1 association as they reposition toward nuclear interiors for transcriptional upregulation. Subsequently, the replication timing of these loci also switches from late to early upon differentiation [40]. Transcription activator-like effector nucleases (TALENs) fused to a VP64 activation domain (tetramer of VP16 transcriptional activator) targeted to *Ptn*, *Sox6*, and *Nrp1* promoters were shown to induce chromatin de-condensation, nuclear relocation, and transcriptional activation [33], which was sufficient to change replication timing of these loci from late to early S-phase. Nevertheless, spatial constraints like TADs or chromosomal territories restrict the capacity of a locus to venture into three-dimensional nuclear space (Figs 1A, 1B and 2A).

The regulation of vertebrate Homeotic complex D (*HoxD*) is unique in many ways. It consists of up to 13 homeobox gene paralogs that follow collinearity both in organization as well as expression along the developing anterior-posterior body axis, and is thus spatiotemporally regulated [41,42]. Numerous enhancers within and outside the complex activate expression of Hox genes in a sequential manner along the anterior (3′) to posterior (5′) side of the complex. This regulation is dependent upon the sequential opening of chromatin along the complex and subsequent reorganization of its promoter–enhancer loops, which, in turn, are regulated and maintained by Polycomb repressive complex machinery [43,44]. This ensures that cognate enhancer–promoter interactions are established, while non-cognate enhancers in the vicinity remain in a closed chromatin state (Fig 2B). During the undifferentiated stage, the entire *HoxD* complex remains in a single TAD. Through differentiation, it is partitioned into two TADs, and a part of the locus transitions between these two TADs, depending upon cell-type identity [45]. Insulators are thought to play a major role in controlling this spatiotemporal opening of the complex. Placing these observations in perspective along with the previously mentioned TAD studies suggests that the cell-type-specific transcriptional program is associated with the modulation of minor enhancer–promoter loops within major chromatin folds of TADs [28].

## What Determines Cognate Enhancer–Promoter Interactions?

Considering that enhancer sequences provide coordinates for spatial and tissue-specific gene expression, it is imperative to understand what brings enhancers to their cognate promoters. Here, we describe the roles of: (1) looping factors, (2) gene regulatory elements, and (3) non-coding RNAs in defining specificity for enhancer–promoter interactions. For more details see [46].

### 1) Looping factors

Proteins that enable chromatin looping are important determinants of enhancer–promoter interactions. In vertebrates, these include proteins such as CTCF and cohesin-associated proteins. The importance of these factors for establishing enhancer–promoter recognition has been shown in several studies. One such example is a study that characterized DNaseI hypersensitive sites (DHS) in the Igf2-H19 locus that are differentially methylated on maternal and paternal alleles. The methylation status of these sites was shown to affect the binding of CTCF, which assists in allele-specific looping of DHS to the *Igf2* promoter and, subsequently, to reinforce these loops, allowing for stable transcription [47]. In another study carried out on CD^+^ CD8^+^ double-positive thymocytes, enhancer clustering was shown to be mediated by cohesin [48]. In cohesin-deficient cells, an active enhancer mark of H3K27ac was still maintained, but proximal genes were misregulated, which is attributed to the loss of enhancer clustering and, hence, non-cognate promoter interactions. Thus, maintaining the looping interaction is thought to be crucial for regulation.

The globin gene cluster has been thoroughly investigated for its looping interactions, since its disruption can lead to various “hemoglobinopathies.” The LIM-domain binding-1 factor (Ldb1) protein binds to both the beta-globin locus control region (LCR) enhancer and promoter, which are 40 kb apart, and mediates long-range interaction by its self-associating domain (SA domain) [49]. The globin cluster LCR activates the gamma-globin gene during fetal development, and during adulthood, the LCR transitions to activate the beta-globin gene while gamma-globin is silenced. In adult mouse primary erythroblasts, the repressed gamma-globin gene was shown to be re-activated, when its promoter was targeted by designed zinc fingers fused with the SA domain of Ldb1. This forced looping also resulted in concomitant loss of beta-globin transcription, as its promoter no longer could contact the LCR [50]. These results suggest that interactions mediated by looping factors not only help to overcome the repressive chromatin environment, but also are crucial for the specificity of enhancer–promoter interactions.

### 2) Gene regulatory elements

The molecular determinants that bring genes to the same transcriptional foci include the gene regulatory elements along with transcription factors, co-factors, and chromatin modifying complexes that bind to them. For example, it was shown that ectopic insertion of the beta-globin LCR could upregulate the expression of neighboring genes [51]. In addition, insertion of the human LCR in mouse chromosome 8 can contact and activate gene expression of the endogenous *Hbb-b1* beta-globin gene on chromosome 7, albeit in a few cells [52]. The ectopic LCR interacted more frequently with loci that were nearby and that were co-regulated by Kruppel-like factor 1 (erythroid) (*EKLF1*) and GATA binding protein 1 (*Gata1*) transcription factors. This suggests that loci with coordinated gene expression are co-regulated by similar transcription factors that may result in their co-localization. Transcription factors and chromatin remodelers that bind to gene regulatory elements determine enhancer–promoter specificity by providing biochemical compatibility to the interacting loci (Fig 2C) [46].

Insulators have also been implicated in maintaining cognate enhancer–promoter interactions (Fig 2D) [53]. One such example is a central transition zone that topologically regulates the coordinated expression of two flanking genes, transcription factor AP-2 gamma (*Tfap2c*) and bone morphogenetic protein 7 (*Bmp7*) [54]. Mouse recombineering experiments demonstrated that disturbing the spatial organization of this region results in reallocation of enhancers and, hence, perturbed tissue-specific gene expression. Essentially, this transition zone helps in maintaining cognate enhancer–promoter interaction, the molecular mechanism of which is still not well understood but is thought to involve insulator elements that maintain distinct regulatory zones.

### 3) Non-coding RNAs

Enhancers often transcribe into RNA, termed eRNAs [55]. Studies have shown that eRNAs are non-polyadenylated and result from bidirectional RNAPolII transcription from active enhancer regions [55–57]. A comprehensive analysis of enhancers in the human genome found bi-directional, unspliced novel transcripts from tissue-specific active enhancers across different cell types [57]. Genome-wide p53 binding studies characterized a subset of p53-dependent enhancers that produce eRNA. Ectopically tethering these eRNAs close to the gene promoter was shown to enhance transcription. Conversely, knocking down eRNAs with small interference RNA (siRNA) results in loss of p53 binding of these enhancers and compromised enhancer function [58]. In another study, time axis dynamics of eRNA transcription on breast cancer cells treated with estrogen showed that eRNA transcription is positively correlated with active enhancer signatures and enhancer looping [59,60]. Combined, these studies suggest that eRNAs could play a crucial role in activating gene promoters.

In an enhancer–promoter looping model, eRNAs could play a role in loop stabilization (Fig 2E). Supporting this is a recent experiment that analyzed eRNA transcription from androgen receptor binding sites that are upregulated upon the binding of transcription factor forkhead box A1 (*FoxA1*). Knockdown of *FoxA1* reduced androgen receptor binding and eRNA transcription. This also resulted in the recruitment of the mediator subunit 12 (Med12) that facilitated enhancer looping interaction with a new set of promoter targets [61,62], indicating that eRNA transcription is crucial for the cognate enhancer–promoter interactions. Long noncoding RNAs (lncRNAs) have also been shown to be involved in mediating enhancer–promoter interactions. One such example are two lncRNAs, PRNCR1 and PCGEM1, which were shown to bind to the androgen receptor and increase its ability to selectively bind to specific enhancers and promoters in prostate cancer cells [63]. However, the mechanisms of noncoding RNA targeting still remain unclear. Future studies may shed more light on whether eRNAs and lncRNAs provide sequence specificity for the cognate interactions or if they assist in the transcriptional assembly at promoter regions with the help of RNA binding factors. This could be done by RNA knockdown or overexpression followed by chromatin conformation analyses that will assess changes in looping.

## Alterations in Enhancer–Promoter Interactions and Human Disease

**Fig 3:**
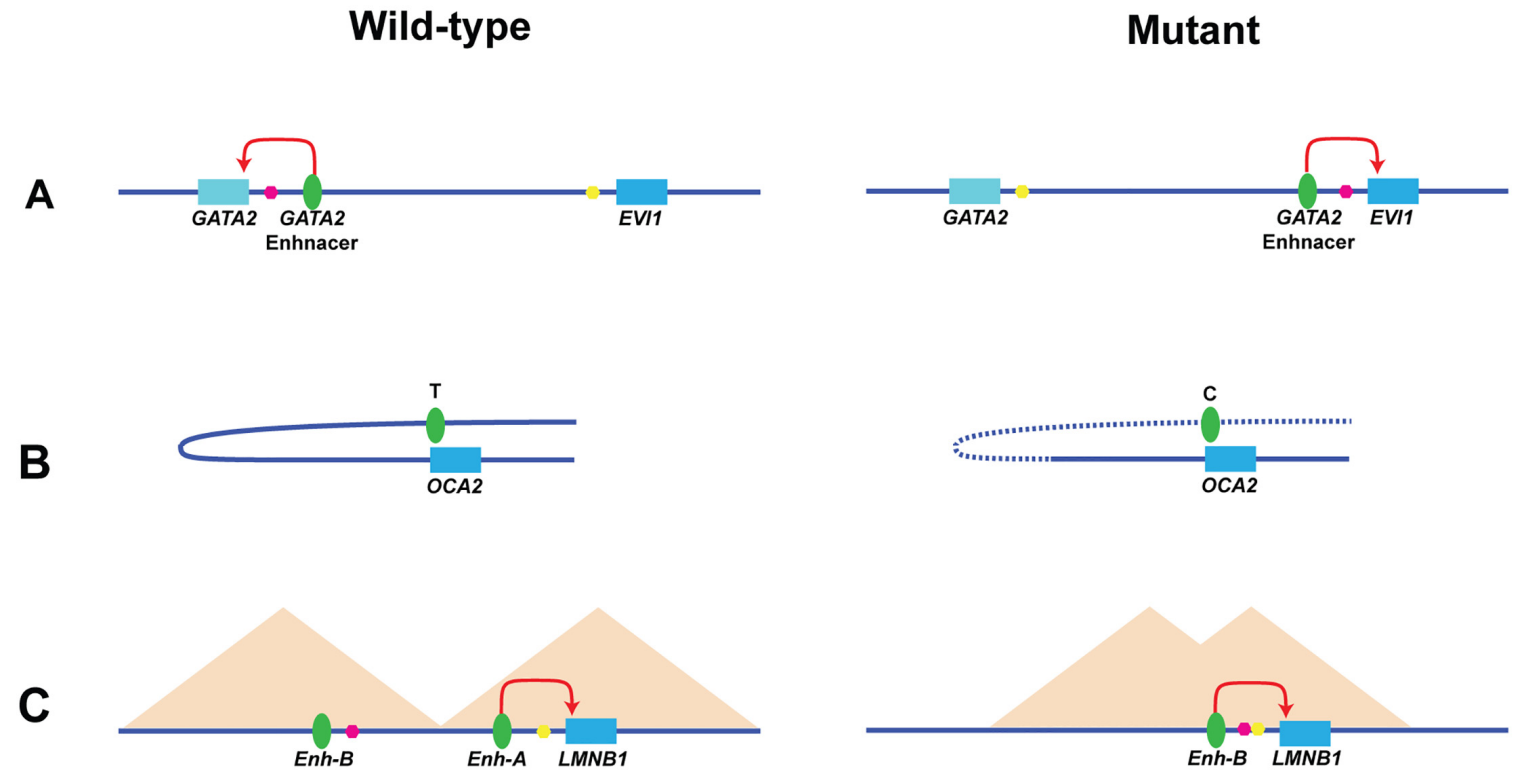
Enhancer–promoter interactions and human disease. (A) An inversion between GATA binding protein 2 (*GATA2*) and ecotopic viral integration site 1 (*EVI1*) repositions a *GATA2* enhancer (green oval) near *EVI1*, leading to its ectopic expression while reducing *GATA2* expression [65]. Inversion breakpoints are depicted by magenta and yellow dots. (B) The T-allele of rs12913832, which resides within an enhancer (green oval) of the oculocutaneous albinism II (*OCA2*) gene, leads to a stronger enhancer–promoter looping interaction versus the C allele, as observed by 3C [66]. (C) A 660 kb deletion that removes a TAD boundary and enhancer-A (Enh-A) leads to enhancer B (Enh-B) adoption by lamin B1 gene (*LMNB1*), leading to its misexpression and, subsequently, autosomal dominant adult-onset leukodystrophy (ADLD) [67]. Deletion breakpoints are depicted by magenta and yellow dots.

Translocations or inversions of genes or enhancers can lead to abnormal enhancer–promoter interactions and subsequent phenotypes, including human disease. For example, an inversion of the *SHH* locus in a patient with features of a holoprosencephaly spectrum caused *SHH* to adopt a different limb enhancer in its new location, leading to its ectopic expression and subsequently causing various limb malformations [64]. Taking it even a step further, a common inversion in human chromosome 3 in leukemia patients between GATA binding protein 2 (*GATA2*) and ecotopic viral integration site 1 (*EVI1*) repositions a *GATA2* enhancer near *EVI1* leading to its ectopic expression while reducing *GATA2* expression [65], thus achieving a “two for the price of one” tumorigenesis affect (Fig 3A).

Nucleotide changes in enhancers or promoters that prevent them from interacting could potentially also lead to human disease and other phenotypes. One such example is a human pigmentation-associated single nucleotide polymorphism (SNP), rs12913832, which is located in a postulated enhancer of oculocutaneous albinism II (*OCA2*) gene. Using 3C, a strong interaction between the enhancer and the *OCA2* promoter was observed for the T pigmentation-associated allele versus a weak interaction for the C unassociated allele (Fig 3B) [66]. However, it is important to keep in mind that not all promoter or enhancer nucleotide changes would lead to phenotypes by looping disruption. For example, looping between the c-MYC promoter and an enhancer that contains a cancer-associated SNP, rs6983267, was shown to occur equally in both alleles, and the phenotype is thought to be driven by an increase in enhancer activity due to differential transcription factor 4 (TCF4) binding [68]. Another example is the sonic hedgehog (*SHH*) limb enhancer, in which mutations in humans, mice, dogs, cats, and chickens were shown to lead to various limb malformations [69,70]. Interestingly, removal of this enhancer in mice, which leads to a limb truncation phenotype, did not disrupt looping of this region to its promoter, but was shown to disrupt looping out of the chromosomal territories [71]. This observation suggests that enhancers could also have an important role in looping interactions that are not associated with the promoter.

## TADopathies: TAD Boundary Abnormalities and Human Disease

TAD alteration can also be associated with human disease. One such example is a 660 kb deletion in a patient with autosomal dominant adult-onset leukodystrophy (ADLD). This deletion leads to a loss of a TAD [67] and causes enhancer adoption, whereby enhancers that in a normal setting would not regulate the lamin B1 gene (*LMNB1*) now lead to its misexpression and, subsequently, ADLD (Fig 3C).

Another example of TAD boundary disruption that leads to human disease is the human chromosome 2q35-36 locus, which is associated with several different limb malformations in humans [72]. Investigating the TAD organization of this locus that harbors the genes *WNT6*, *IHH*, *EPHA4*, and *PAX3* revealed that it is structurally divided into three independent TADs: *WNT6*/*IHH*-TAD, *EPHA4*-TAD, and *PAX3*-TAD. Chromosomal aberrations that include deletions, inversions, and duplications that alter the TAD boundaries were shown to cause a variety of limb malformation phenotypes in humans. Analysis using 4C-seq of both patient-derived fibroblasts and mice engineered to model these aberrations showed that the deletions, duplications, and inversions encompassing the TAD boundaries of this locus lead to ectopic expression of surrounding genes. Deletions designed to spare the TAD boundaries but that remove the majority of the sequence that is affected in the patients did not result in any overt limb phenotype. This suggests that the loss of spacing or distance between enhancer and promoter does not cause the limb phenotype, but rather, the loss of TAD boundaries does.

These results provide strong evidence that disruption of TAD boundaries as a result of chromosomal aberrations could be a cause of human disease, termed here as TADopathies. The functional components that make up these TAD boundaries are still not well understood. TADs are likely to be maintained by a variety of nuclear proteins. Mutations in these proteins that affect their binding could also result in altered gene expression and various human diseases.

## Future Directions

Despite numerous studies and technological advancements, we still do not have a comprehensive understanding of the factors that mediate chromatin interactions between loci. Techniques investigating structural organization of chromatin need to be integrated with finite transcriptional measurements. Imaging studies probing long-range interactions yield only a subset of nuclei where co-localization can be observed. Biochemical methods such as Hi-C generate genome-wide interactions on the basis of pairwise enrichment in a population of cells. Recently, single-cell analytical approaches have been developed to bridge this gap between microscopic and biochemical methods [73,74]. A single-cell Hi-C study on ten T-helper cells identified specific chromosome territories with active transcriptional domains at their interfaces, corroborating that active loci relocate when transcribed [75]. Another caveat with current approaches is that they only provide snapshots of a certain temporal stage. Recently, a study that used a combination of several techniques that include light sheet microscopy and single molecule tracking allowed for the live, single-cell characterization of Sox2-mediated 3-D enhancer clustering [76]. Future developments that can provide for more dynamic approaches combined with microscopy to assay these looping interactions in real time could rapidly advance this field.

There could be several additional enhancer–promoter interaction-associated factors that have yet to be identified. Numerous additional structural proteins, gene regulatory elements, or even DNA sequences that are involved in these interactions could exist. For example, recent computational work suggests that DNA G-quadruplexes (a motif of three to four consecutive guanines) could be involved in enhancer–promoter interactions [77]. Studies that perturb these specific factors and analyze their effect on chromatin interactions will greatly assist in obtaining a better understanding of how enhancer–promoter specificity is achieved. Similar to the zinc finger Ldb1 SA domain globin looping disruption [50], experiments that take advantage of nuclease-deficient Cas9 (dCas9) fusions followed by interaction and phenotypic analyses, for example, could be used to obtain a better understanding of the consequences of these disruptions. Combined, these experiments could increase our understanding of the functional consequences of mutations within these factors and regulatory elements.
